# Divergent roles for complement components C3 and C4 in controlling *Klebsiella pneumoniae* gut colonization and systemic dissemination

**DOI:** 10.1128/mbio.03416-25

**Published:** 2026-02-11

**Authors:** Juan D. Valencia-Bacca, Jamie E. Jennings-Gee, Noah A. Nutter, Alexis E. Adams-Sims, Abigail A. Hegarty, Hope L. Nzuki, Ravinder K. Nagpal, M. Ammar Zafar, Karen M. Haas

**Affiliations:** 1 Department of Microbiology and Immunology, Wake Forest University School of Medicine 12279, Winston-Salem, North Carolina, USA; 2 Department of Health, Nutrition, and Food Sciences, College of Education, Health, and Human Sciences, Florida State University 7823, Tallahassee, Florida, USA; 3 Department of Microbiology and Immunology, Emory University School of Medicine 12239, Atlanta, Georgia, USA; Yale School of Medicine, New Haven, Connecticut, USA

**Keywords:** *Klebsiella pneumoniae*, complement deficiency, intestinal colonization, antibiotic resistance

## Abstract

*Klebsiella pneumoniae* is an escalating public health threat driven by the emergence of antibiotic-resistant and hyper-encapsulated strains that spread systemically from the gut. The immune defenses preventing gut colonization and dissemination remain poorly defined. Here, we identify distinct and context-dependent roles for complement proteins C3 and C4 in host defense following *K. pneumoniae* infection. Following gut colonization, the levels of C3 and C4 significantly increase, and C3/C4 deposition is found on *K. pneumoniae* within the gut. *In vitro*, complement from rabbit, mouse, and human sources induced C3b deposition on *K. pneumoniae* grown under gut-mimetic conditions, even when C4-dependent pathways were inhibited. In addition to promoting opsonization, C3 was found to be critical for recruiting myeloid cells to the gut and for preventing lethal systemic spread. Depletion of systemic C3 revealed mucosal-derived C3 controls *K. pneumoniae* gastrointestinal colonization, whereas systemic C3 is essential for limiting fatal dissemination. In contrast, C4 is dispensable for controlling gastrointestinal colonization, dissemination, and myeloid recruitment under conditions of natural acquisition. However, C4 is critical for reducing gut burden and systemic disease following antibiotic-induced dysbiosis and supercolonization with antibiotic-resistant *K. pneumoniae*. Collectively, these findings reveal a dual-layered immune strategy: C3-driven opsonization and phagocyte recruitment, independent of C4, provide a mechanism for rapid containment of *K. pneumoniae* gastrointestinal colonization and dissemination under baseline conditions, whereas C4 becomes critical for controlling high bacterial burdens associated with antibiotic resistance. This work advances our understanding of complement-dependent mucosal immunity and identifies potential targets to prevent gut-to-bloodstream transition of this formidable pathogen.

**IMPORTANCE:**

*Klebsiella pneumoniae*, a major public health threat, resists antibiotics and can spread from the gut to the bloodstream, causing severe infections. Our study reveals how the immune system uses complement proteins C3 and C4 to block this spread. C3 limits bacterial growth in the gut through two potential mechanisms: (i) coating *K. pneumoniae* with fragments that signal bacteria-eating phagocytic cells to destroy it and (ii) recruiting more phagocytes into the gut. C3 also helps clear bacteria that escape into the blood. However, when antibiotic-resistant strains overgrow, C3 alone is insufficient. In these cases, C4 becomes critical, likely by enhancing C3’s ability to tag bacteria for elimination. This two-layered defense highlights new immune pathways that could be targeted to prevent bloodstream infections, especially in vulnerable patients or those colonized with drug-resistant bacteria. These insights open doors to innovative strategies against life-threatening *Klebsiella* infections.

## INTRODUCTION

*Klebsiella pneumoniae* (*Kpn*) is well-recognized as one of the leading causes of antimicrobial-resistant opportunistic infections (1). *Kpn* can silently colonize the gastrointestinal (GI) tract of healthy individuals without causing clinical manifestations (2–4). Nonetheless, prolonged presence of virulent strains may contribute to inflammation-driven GI diseases, including cancer (5–8). Moreover, GI colonization is linked to a heightened risk of subsequent life-threatening extraintestinal infections (9, 10). In particular, hypervirulent *Kpn* strains which typically exhibit a hypermucoviscous phenotype comprising K1/K2 capsule types possess an enhanced ability to cross the intestinal mucosal barrier (11, 12). *Kpn* requires a combination of adhesion factors, stress response genes, and genetic regulatory networks to translocate from the GI tract and establish extraintestinal infections (13)—a process that may entail transcellular invasion without disrupting tight junctions or epithelial integrity (14). Identifying host defense mechanisms that protect against *Kpn* colonization, translocation, and dissemination is critical for devising strategies to protect against *Kpn* infections threatening human health.

Host defense against bacterial colonization in the gut relies on multiple factors, including the microbiota, mechanical barriers, and innate and adaptive immune responses (15). Among these, innate immunity plays a pivotal role as the first line of defense, detecting microbial invaders through pattern recognition receptors and rapidly initiating phagocytic and inflammatory responses to restrict the expansion and translocation of harmful, non-commensal microorganisms (16). Despite this, the role of the complement system—a key element of innate immunity—in protecting against intestinal infections remains unclear. While the liver is the main source of complement proteins (17, 18), non-hepatic sources, such as immune cells, intestinal epithelial cells, and colonic stromal cells, also synthesize these components (19–21). The function of the complement system in the gut has been largely overlooked, partly due to the limited detection of complement components in the healthy GI lumen (20). This perception is further reinforced by the prominent role of secretory IgA in gut immunity, which, despite its effectiveness in promoting non-inflammatory clearance of pathogens and maintaining mucosal homeostasis, has a limited capacity to activate complement (22). As a result, few studies have specifically investigated the role of complement in gut colonization and subsequent systemic dissemination, leaving significant gaps in our understanding of its contribution to early-stage host defense. However, a recent study by Wu et al. showing a critical role for the C3-dependent alternative pathway in providing protection against *Citrobacter rodentium* GI infection in mice has prompted interest in this area (21).

Reports on the susceptibility of *Kpn* strains to complement-mediated killing and clearance are variable. While serum-sensitive strains activate both the classical and alternative complement pathways *in vitro*, leading to C3b deposition and subsequent lysis via membrane attack complex (MAC) formation (23), serum-resistant strains evade MAC-mediated lysis primarily due to a thick capsule and/or extended O-antigen polysaccharide chains in lipopolysaccharide (LPS), which can redirect C3b deposition away from the bacterial membrane (24, 25). Notably, in clinical isolates lacking the LPS O antigen, the capsular type has been shown to be a key determinant of complement resistance (26). Interestingly, a recent study demonstrated that K23 capsule-expressing *Kpn* was cleared by a C3-independent mechanism involving liver-resident Kupffer cells (27). Mouse models of *Kpn* lung infection investigating C3 effects using the virulent K2 capsule-type KPPR1 (ATCC 43816) *Kpn* strain have shown contradictory roles, with one study showing increased splenic dissemination in C3^−/−^ mice (28), whereas another showed no differences in survival relative to wild-type (WT) mice (29). Thus, the role of complement in controlling systemic *Kpn* infection is contradictory. Furthermore, the role of complement in controlling *Kpn* gut colonization and subsequent systemic dissemination is completely unknown. Herein, we demonstrate context-dependent roles for C3 and C4 in controlling *Kpn* GI-associated pathogenesis.

## RESULTS

### Complement component C3, but not C4, is required to limit *Kpn* burden in the gut following natural acquisition

**Fig 1:**
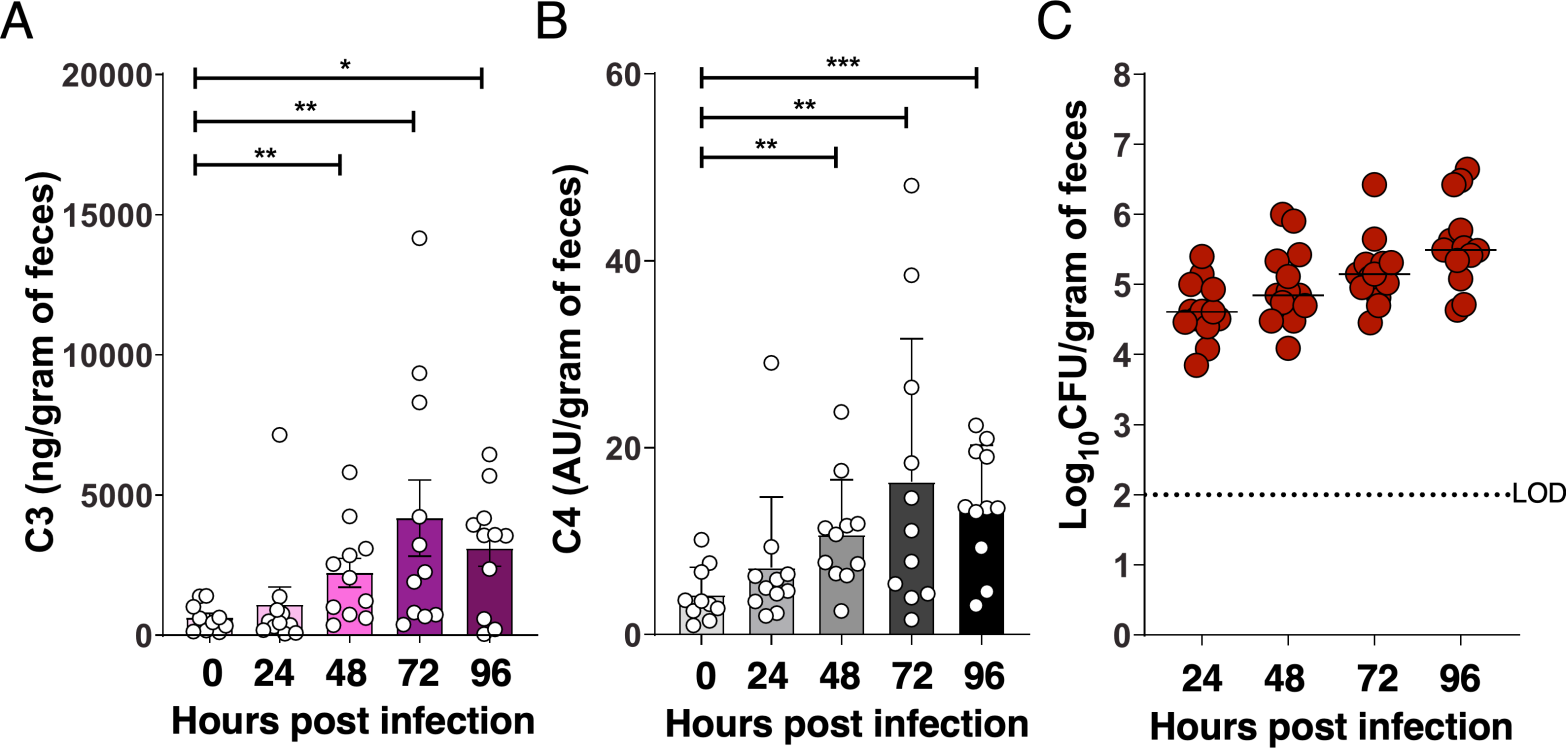
Fecal C3 and C4 levels increase following GI colonization with *Kpn*. (**A, B**) C3 (**A**) and C4 (**B**) levels measured by ELISA in fecal samples from C57BL/6J WT mice before infection and at 24, 48, 72, and 96 h following oral inoculation with *Kpn* strain KPPR1S (~10⁶ CFU). (**C**) Corresponding fecal shedding in mice colonized with KPPR1S. Each symbol represents an individual mouse (*n* = 13 per group). Statistical significance was assessed by one-way ANOVA with Bonferroni *post hoc* test (**A, B**). *P < 0.05, **P < 0.01, ***P < 0.001, and ****P < 0.0001.

Oral inoculation of *Kpn* strain KPPR1S (ATCC 43816, ST493, K2 serotype) leads to robust GI colonization in WT mice with no noticeable clinical effects (30). However, it is possible that *Kpn* colonization induces subclinical inflammation. We therefore measured C3 and C4 complement protein levels in fecal samples from naïve and orally inoculated C57BL/6 WT mice (Fig. 1A and B), along with fecal shedding (Fig. 1C). Consistent with previous studies, fecal samples from naïve mice exhibited low basal or undetectable levels of C3 and C4 (21). However, oral inoculation with *Kpn* led to significant increases in the levels of fecal C3 (>5-fold; Fig. 1A) and C4 (>3-fold; Fig. 1B) at 48, 72, and 96 h. These findings, along with increases in fecal lipocalin-2 (31), were corroborated in independent experiments (Fig. S1A through C) and support the presence of an inflammatory response in the gut following infection. Furthermore, when we orally colonized mice with GFP^+^ KPPR1S *Kpn* cells for 4 days and supplied streptomycin water during the final 24 h to induce supercolonization and enable detection by flow cytometry, GFP^+^ KPPR1S isolated from the feces had detectable levels of C3b and C4b deposition (Fig. S1D and E).

**Fig 2:**
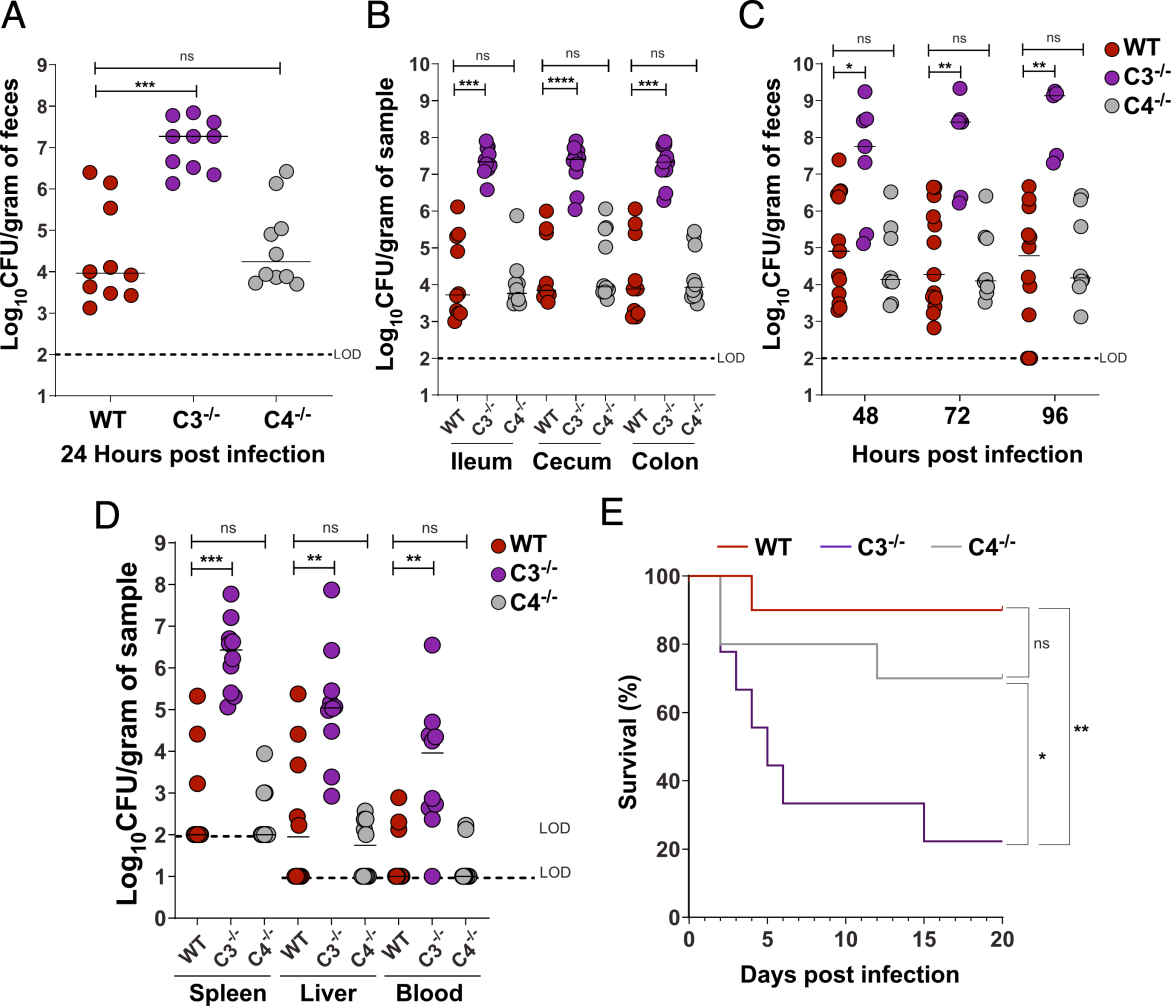
C3, but not C4, controls *Kpn* gut burden, systemic dissemination, and survival following oral inoculation. (**A**) Fecal shedding in mice colonized with KPPR1S 24 h post-infection. (**B**) Bacterial densities in the ileum, cecum, and colon at 24 h. (**C**) Fecal shedding at 48, 72, and 96 h. (**D**) Bacterial burden in spleen, liver, and blood at 24 h. (**E**) Kaplan-Meier survival curve indicating survival following oral KPPR1S inoculation. In panels A–D, each circle represents an individual mouse. Similar results were obtained in two independent experiments with four to five mice per genotype. Pooled data are shown. Dotted lines indicate the limit of detection. Statistical significance was assessed by Kruskal-Wallis with Dunn’s *post hoc* test (**A, B**); *n* ≥ 9; (**C**), *n* ≥ 7; (**D**), *n* ≥ 9. *P < 0.05, **P < 0.01, ***P < 0.001, ****P < 0.0001; ns, not significant . Statistical significance was assessed using the log-rank (Mantel-Cox) test in panel **E**, *n* ≥ 9.

Given the above findings, we next determined whether C3 and/or C4 influence bacterial burden in the GI tract by evaluating *Kpn* GI colonization in WT, C3^−/−^, and C4^−/−^ mice. At 24 h post-inoculation, C3^−/−^ mice, but not C4^−/−^ mice, exhibited a significantly higher bacterial burden (1,000-fold increase) in feces relative to WT mice (Fig. 2A). At 24 h post-challenge, recovered CFUs were also significantly increased (1,000-fold) at all sites sampled along the GI tract (ileum, cecum, and colon) of C3^−/−^ mice, whereas C4^−/−^ burden was similar to that of WT mice (Fig. 2B). This striking phenotype persisted up to 96 h post-challenge (Fig. 2C). As expected, C3 was not detected in fecal pellets of C3^−/−^ mice but was present at WT levels in infected C4^−/−^ mice (Fig. S1F and G), consistent with findings for circulating levels in WT and C4^−/−^ mice (32). Nonetheless, we did not detect C4 in C3^−/−^ fecal samples, in contrast to the normal levels of circulating C4 found in C3^−/−^ mice (32). Thus, C3, but not C4, is critical for limiting *Kpn* intestinal burden following oral challenge.

### Complement component C3, but not C4, is required to limit *Kpn* extraintestinal dissemination and death following natural acquisition

Molecular epidemiological studies suggest *Kpn* gut colonization is a marker for subsequent invasive disease (9, 10). We therefore assessed the presence of *Kpn* in the liver, spleen, and blood of WT, C3^−/−^, and C4^−/−^ mice. At 24 h post-inoculation, approximately half of the WT and C4^−/−^ mice harbored *Kpn* in the liver (Fig. 2D), and to a lesser extent in the spleen and blood. However, all C3^−/−^ mice were colonized with *Kpn* in the liver, spleen, and blood, with bacterial densities ~1,000-fold over that found in WT and C4^−/−^ mice. Consequently, the majority of C3^−/−^ mice succumbed to infection, in contrast to WT and C4^−/−^ mice (Fig. 2E). Thus, besides limiting *Kpn* GI colonization and extraintestinal burden following oral *Kpn* colonization, C3, but not C4, is required for survival after dissemination.

### *Kpn* is resistant to complement-mediated lysis under *in vitro* gut-mimicking growth conditions but remains sensitive to C3b deposition in a C4-independent manner

**Fig 3:**
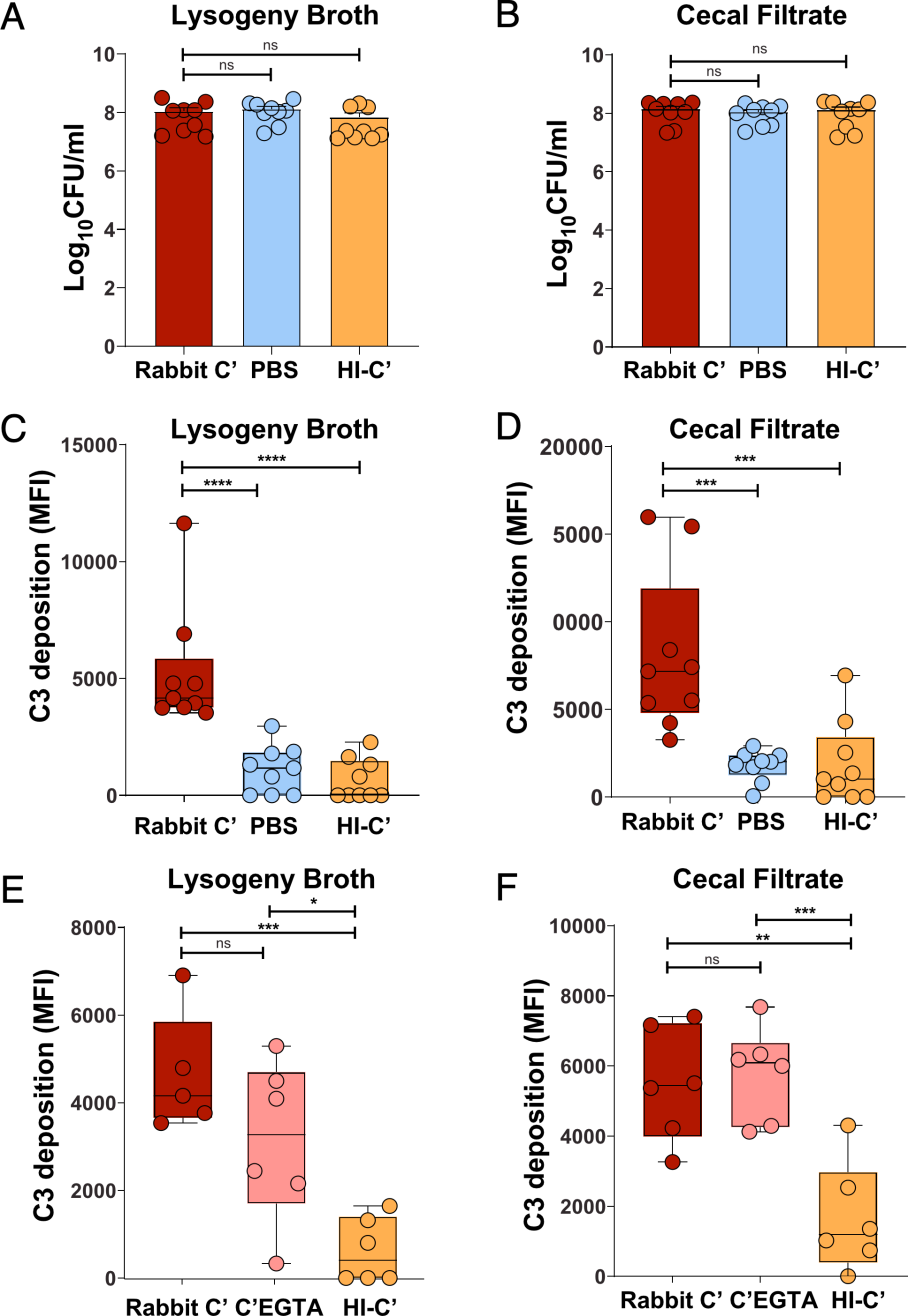
*In vitro* C3b deposition on *Kpn* occurs in the presence of EGTA. (**A, B**) *Kpn* KPPR1S was incubated for 1 h at 37°C in LB (**A**) or cecal filtrate (**B**) in the presence of 10% rabbit complement, heat-inactivated complement (HIC), or PBS. Recovered CFU were assessed, and significance was determined using the Kruskal-Wallis test with Dunn’s *post hoc* test. (**C–E**) C3b deposition on KPPR1S grown in LB or cecal filtrate was analyzed by flow cytometry after a 1 h incubation at 37°C. (**C–F**) Flow cytometry quantification of C3b deposition on KPPR1S in LB (**C, E**) or cecal filtrate (**D, F**) with 10% rabbit complement, EGTA-treated complement, or HIC. (**A–D**) Pooled results from three independent experiments. (**E, F**) pooled results from two to three independent experiments. Statistical analysis: one-way ANOVA with Bonferroni *post hoc* test (*n* ≥ 5 per group). *P < 0.05, **P < 0.01, ***P < 0.001, ****P < 0.0001; ns, not significant.

The inability of C3^−/−^ mice to control *Kpn* gut burden could be due to defects in complement-mediated opsonophagocytosis and/or the ability of the complement system to directly kill gram-negative bacteria through MAC formation. To investigate potential mechanisms by which C3 contributes to reduced gut burden, we assessed complement-mediated effects on *Kpn* grown in either gut-mimetic media (cecal filtrate) or lysogeny broth (LB). Bacteria were incubated with 10% rabbit complement for 30 or 60 min, and survival was determined. As shown in Fig. 3A and B, KPPR1S remained completely resistant to complement-mediated killing regardless of whether it was cultured in LB or cecal filtrate, with no significant reduction in CFU relative to 10% heat-inactivated complement or PBS alone, consistent with a previous report (25). In contrast, an isogenic capsule-deficient strain (Δ*wcaJ*) (30) was highly susceptible to complement-mediated killing, with 10% rabbit complement reducing recovered CFU 1,000-fold (Fig. S2A and B). Heat-inactivated complement did not affect bacterial survival relative to PBS, confirming that the killing was specifically mediated by active complement components.

**Fig 4:**
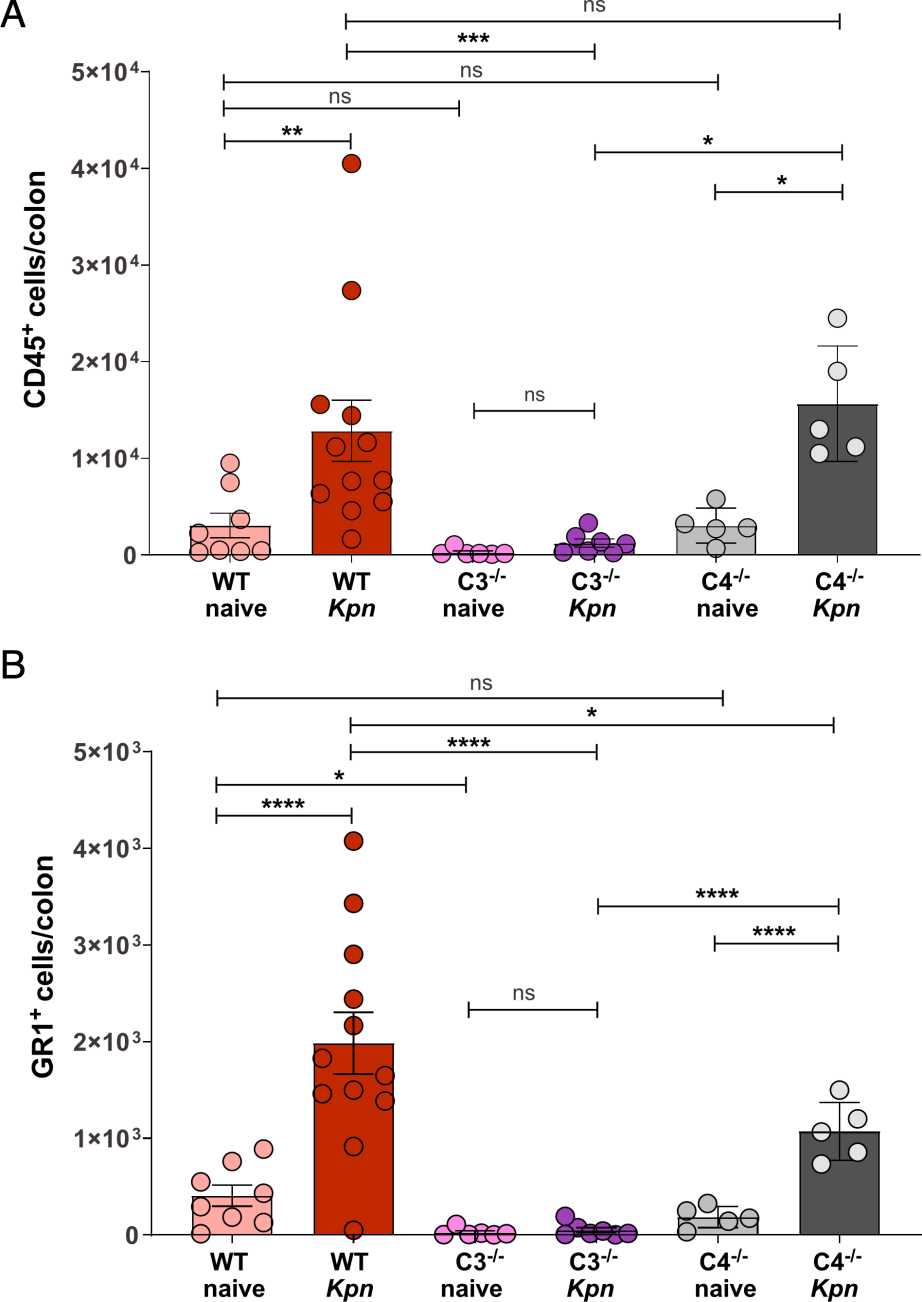
Colonic leukocyte and myeloid cell infiltration following oral *Kpn* inoculation. (**A, B**) Flow cytometry detection of CD45^+^ leukocyte (**A**) and GR1^+^ myeloid (**B**) cells in the colons of naïve control mice and at 48 h post-oral inoculation with KPPR1S. Pooled results from two to three independent experiments, with each circle indicating results from an individual mouse. Statistical outliers associated with sample processing issues and poor colonization were removed in GraphPad (Rout, Q = 1%) in panels A and B. Outliers identified included 1 C3^−/−^ naïve, 1 C3^−/−^ infected, and 1 WT infected mouse. Statistical analysis was conducted using two-way ANOVA (*n* ≥ 5 mice per group). *P < 0.05, **P < 0.01, ***P < 0.001, ****P < 0.0001; ns, not significant.

Although KPPR1S was not killed by 10% active rabbit complement, it induced significant C3b deposition on KPPR1S grown in either LB or cecal filtrate (Fig. 3C and 4D), as measured by flow cytometry (Fig. S2C). In contrast, no C3b deposition was observed in the presence of heat-inactivated complement. Pretreatment of rabbit complement with EGTA, which chelates calcium ions and selectively inhibits the classical and lectin pathways, modestly reduced C3b deposition when *Kpn* was grown in LB (Fig. 3E) but had no detectable effect on C3b deposition for bacteria grown in cecal filtrate (Fig. 3F). These findings suggest that the alternative pathway may be a major driver of C3b deposition under gut-mimicking conditions and may, in part, explain the lack of effect of C4 deficiency on *Kpn* colonization levels. C3b deposition was similarly induced on KPPR1 using fresh mouse sera and commercial Ig-depleted human complement, even in the presence of EGTA (Fig. S2D and E), supporting the potential for the alternative pathway to opsonize bacteria in the gut environment.

### *Kpn* GI colonization induces C3-dependent myeloid cell recruitment into the colon

In addition to supporting C3b-mediated phagocytosis, C3 activation can support leukocyte recruitment into sites of inflammation (33). We assessed leukocyte recruitment into the colon by flow cytometry 48 h after oral inoculation and found a significant increase in CD45^+^ cells in infected WT and C4^−/−^ mice (Fig. 4A; Fig. S3), which was concomitant with a fivefold increase in CD45^+^GR-1^+^SSC^hi^ (Fig. 4B), indicative of myeloid cell recruitment (34). Interestingly, baseline levels of CD45^+^ and GR1^+^ cell numbers were lower in C3^−/−^ than in WT mice, and in contrast to WT and C4^−/−^ mice, we did not observe a significant increase in these cells following oral inoculation in C3^−/−^ mice. Thus, C3, but not C4, is essential for restricting *Kpn* burden and likely does so by inducing C3b deposition and/or supporting phagocyte recruitment to the intestine following infection.

### Modest microbiome differences in C3^−/−^ mice are unlikely to contribute to increased susceptibility to *Kpn*

**Fig 5:**
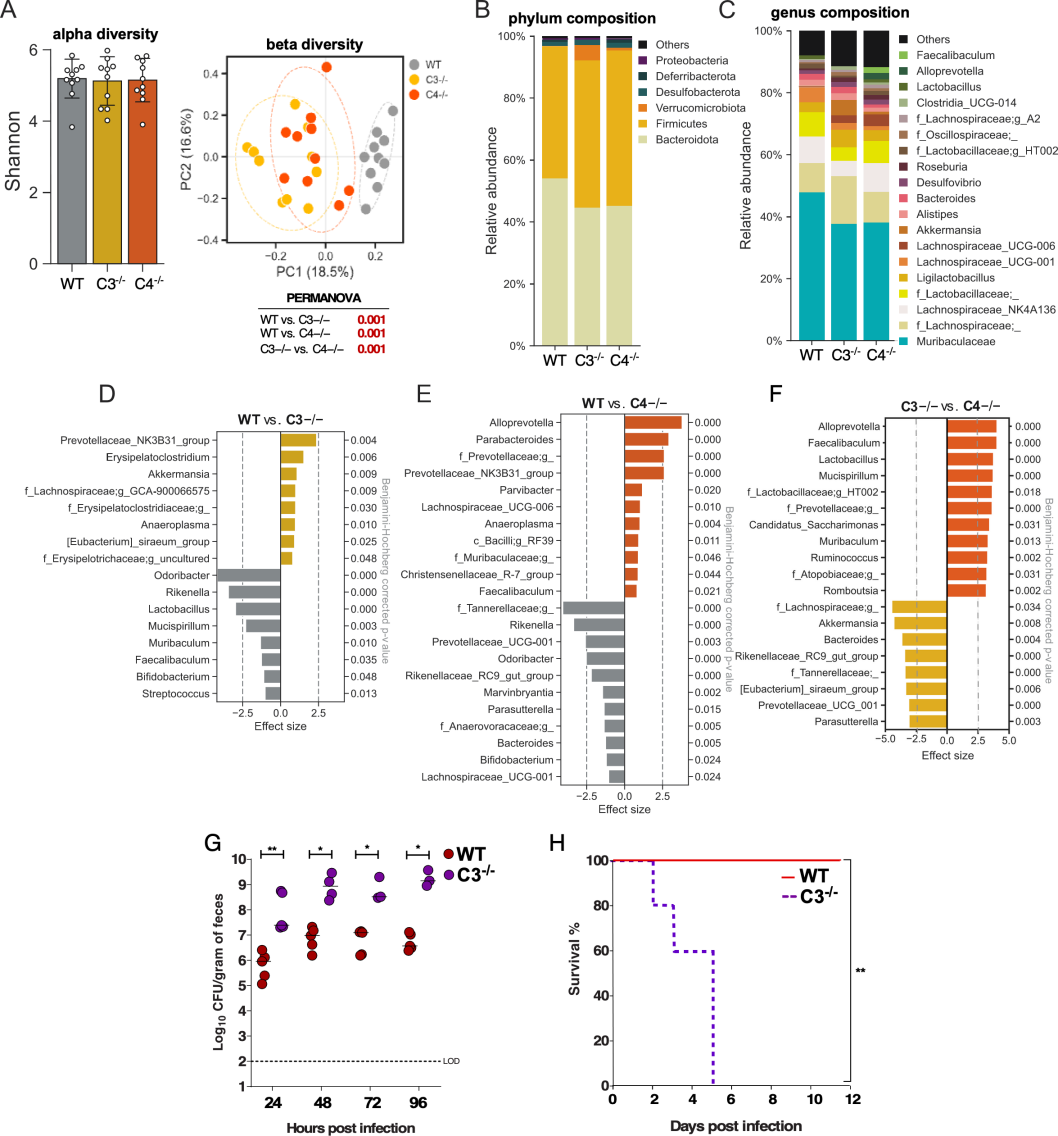
Fecal microbiome analysis for WT, C3^−/−^, and C4^−/−^ mice and KPPR1S infection outcomes in co-housed WT and C3^−/−^ mice. (**A–E**) Microbiome analysis for fecal pellets obtained from 10 male mice from each genotype was performed to assess alpha- and beta-diversity. Similar alpha-diversity among the three groups, but distinct beta-diversity linked to *Lactobacillus* was found. Alpha- and beta- diversity (**A**) and the gut microbiota composition at the level of major bacterial phyla (**B**) and genera (**C**). Linear discriminant analysis (LDA) effect size (LEfSe) analyses showing major features (bacterial taxa) driving differences among genotypes (**D–F**). (**G, H**) WT and C3^−/−^ weanling mice were co-housed for 3 weeks prior to oral inoculation with KPPR1S (**G**). Fecal shedding was measured at 24, 48, 72, and 96 h post-inoculation. Each point represents an individual mouse (*n* ≥ 5 per group). (**H**) Kaplan-Meier survival curve indicating survival following oral KPPR1S inoculation. Statistical analysis: Mann-Whitney *U*-test (**F**) and the log-rank (Mantel-Cox) tests (**H**), *P < 0.05, **P < 0.01, ***P < 0.001, ****P < 0.0001.

To investigate potential differences in the microbiome that could contribute to the above findings, 16S rRNA sequencing was performed on fecal samples from WT, C3^−/−^, and C4^−/−^ mice (10 samples/genotype) born and housed in the same room of our animal facility. Our analysis shows a similar level of alpha-diversity (Shannon index) among the three groups, with slight differences observed in beta-diversity and microbiome composition (Fig. 5A through F). Relative to C3^−/−^ mice, WT and C4^−/−^ mice exhibited increased abundance of *Lactobacillus*—a key contributor to colonization resistance against *Kpn* (35–37). The only other shared increases in WT and C4^−/−^ mice relative to C3^−/−^ mice were *Mucispirillum* and *Muribaculum,* the latter of which is depleted by *Kpn* (38). Based on these results, we co-housed WT and C3^−/−^ weanling mice for 3 weeks to promote microbiota homogenization through shared environment and coprophagy. Despite co-housing, C3^−/−^ mice exhibited significantly increased *Kpn* GI burden and dissemination compared to WT housemates (Fig. 5G and H), indicating that their susceptibility is unlikely to be mitigated by microbiota equilibration. Nonetheless, potential effects of altered *Lactobacillus* levels were further addressed in subsequent experiments using streptomycin treatment, as detailed below.

### Control of *Kpn* GI burden and dissemination in the context of antibiotic-induced supercolonization requires both C3 and C4

**Fig 6:**
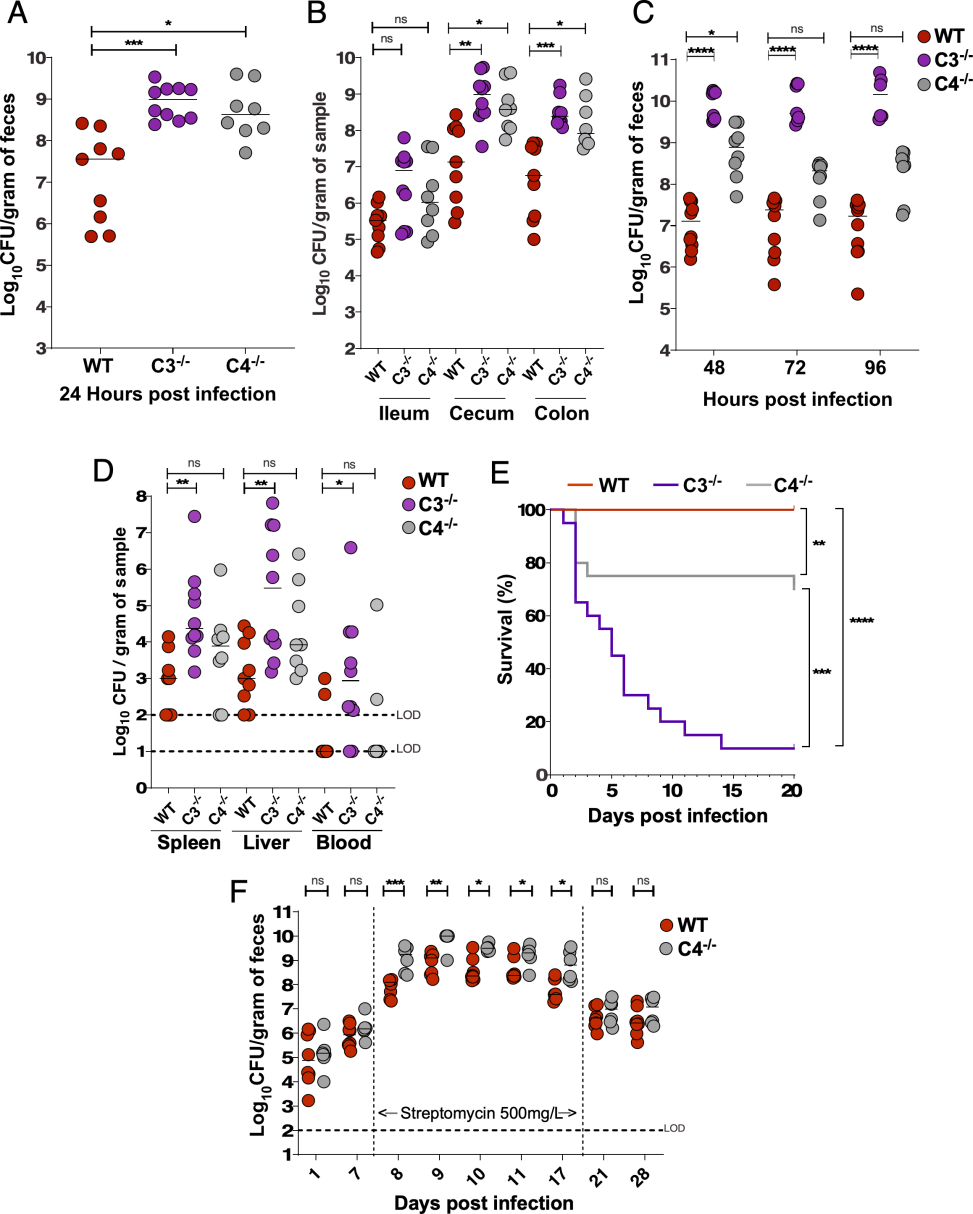
C3 and C4 are both required to control *Kpn* GI burden and limit dissemination and mortality in the context of antibiotic-induced supercolonization. (**A–E**) WT, C3^−/−^, and C4^−/−^ mice were treated with streptomycin (500 µg/mL in drinking water) 3 days prior to intragastric gavage with KPPR1S (10^7^ CFU). (**A**) Fecal shedding of KPPR1S at 24 h post-gavage. (**B**) KPPR1S bacterial loads in the ileum, cecum, and colon at 24 h post-gavage. (**C**) Fecal shedding of KPPR1S at 48, 72, and 96 h post-gavage. (**D**) Systemic dissemination assessed by bacterial burden in spleen, liver, and blood at 24 h post-gavage. (**E**) Kaplan-Meier survival curves of WT, C3^−/−^, and C4^−/−^ mice following KPPR1S oral gavage. (**F**) Fecal shedding of KPPR1S in WT and C4^−/−^ mice following oral inoculation (~10⁶ CFU). Streptomycin (500 mg/L) was introduced in drinking water on day 7 to induce a supershedder phenotype and removed on day 17. Dotted lines indicate the limit of detection. Each circle indicating results from an individual mouse. (**A–E**) Pooled results from two to three independent experiments. Statistical significance was determined using the Kruskal-Wallis test with Dunn’s *post hoc* test (A–D, *n* ≥ 9/group), log-rank (Mantel-Cox) tests (E, *n* ≥ 20), and Mann-Whitney U-test (F, *n* ≥ 8/group). *P < 0.05, **P < 0.01, ***P < 0.001, ****P < 0.0001; ns, not significant.

In clinical settings, prolonged antibiotic therapy is a significant risk factor for developing severe and invasive *Kpn* infections (39). Antibiotic use reduces microbial diversity in the GI tract and facilitates high-level colonization of antibiotic-resistant *Kpn* (40). To investigate the role of complement in *Kpn* GI colonization and dissemination in the context of antibiotic treatment, we employed a broad-spectrum antibiotic treatment model using streptomycin, delivering the bacterial load via orogastric gavage. Streptomycin treatment is well-known to deplete *Lactobacillus* (41) and results in supercolonization by the streptomycin-resistant KPPR1S strain, with colonization levels increased 1,000-fold over that found with oral inoculation in the absence of antibiotic treatment (30). Similar to our results obtained with oral feeding, with antibiotic treatment, C3^−/−^ mice displayed 100- to 1,000-fold greater *Kpn* CFU in fecal shedding, cecum, and colon relative to WT mice (Fig. 6A through C). In contrast to our results with natural colonization, whereby C4 deficiency had no observable effect on *Kpn* gut burden, with antibiotic treatment, C4^−/−^ mice had significantly increased fecal shedding, cecum, and colon CFUs relative to WT mice, and at levels that were comparable to C3^−/−^ mice (Fig. 6A through C). Additional exploratory Dunn’s *post hoc* analyses indicated no significant differences in fecal shedding levels (24–48 h post-infection) or in intestinal tissue burdens between C3^−/−^ and C4^−/−^mice.

Relative to WT mice, C3^−/−^ mice exhibited significantly increased bacterial burden in extraintestinal sites (spleen, liver, blood) at 24 h post-infection (Fig. 6D). A similar trend was observed in C4^−/−^ mice in the liver (*P* = 0.07), suggesting a potential role for C4 in limiting early dissemination (Fig. 6D). Consistent with this, survival was significantly decreased in highly colonized C3^−/−^ (10%) and C4^−/−^ (75%) mice relative to WT mice (100%) (Fig. 6E), highlighting the pivotal role of C3 in controlling systemic dissemination. Notably, direct systemic KPPR1 challenge resulted in similar survival defects, with C3^−/−^ mice exhibiting significantly increased susceptibility relative to C4^−/−^ mice (Table S1), thereby highlighting a more critical role for C3 relative to C4 in limiting fatal dissemination.

To determine whether the significantly increased GI burden in C4^−/−^ mice in the presence of antibiotic treatment was limited to early colonization, we orally infected WT and C4^−/−^ mice with an intact microbiota and then administered streptomycin 1 week post-infection. As shown in Figure 6F, C4^−/−^ mice exhibited WT levels of KPPR1S GI burdens following oral feeding. However, 1 day following streptomycin administration, C4^−/−^ mice exhibited significantly higher *Kpn* levels relative to WT mice. Importantly, once the streptomycin treatment was discontinued, colonization levels in C4^−/−^ mice returned to WT levels (Fig. 6F).

### Depletion of circulating C3 in WT mice has no effect on *Kpn* GI colonization but leads to fatal dissemination

**Fig 7:**
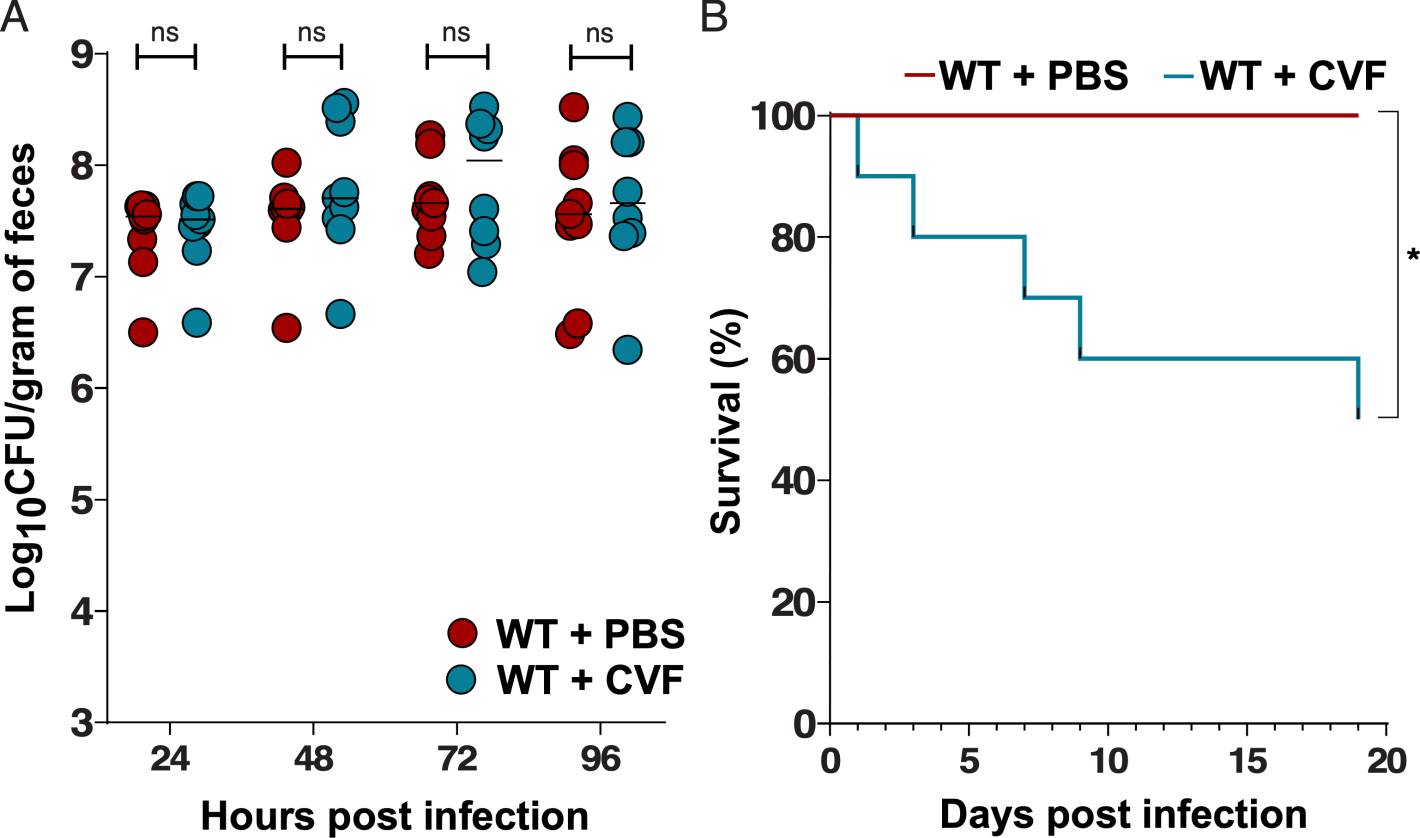
Depletion of circulating C3 in WT mice increases fatal dissemination but has no effect on *Kpn* GI colonization levels. (**A**) Fecal shedding in WT PBS-control and CVF-treated WT mice at 24, 48, 72, and 96 h post-KPPR1S gavage inoculation. (**B**) Kaplan-Meier survival of PBS control and CVF-treated WT mice following oral KPPR1S gavage. Statistical significance was determined using Mann-Whitney U-test (**A**) for bacterial burden (*n* ≥ 9/group) and the log-rank (Mantel-Cox) tests (**B**) for survival (*n* ≥ 9). *P < 0.05, **P < 0.01, ***P < 0.001, ****P < 0.0001; ns, not significant.

To distinguish the roles of systemic versus locally produced C3 in protection against invasive disease, we used cobra venom factor (CVF) to deplete circulating C3 while preserving GI-derived C3 (21). Depletion of systemic C3 activity in WT mice had no effect on *Kpn* burden in the gut (Fig. 7A). However, a significant fraction of CVF-treated WT mice succumbed to bacterial dissemination (Fig. 7B), indicating that circulating complement is critical for controlling systemic infection but is dispensable for regulating bacterial colonization in the GI tract.

### C3 and C4 are required for control of antibiotic-induced supercolonization of classical *Kpn* strain MKP103

Due to genetic heterogeneity of *Kpn* isolates (42), we investigated whether GI colonization with the classical multi-drug-resistant *Kpn* strain MKP103—a derivative of the outbreak strain KPNIH1 (ST258) (43)—is similarly regulated by C3 or C4 in the context of ampicillin treatment. While no differences in colonization were observed at 24 h, MKP103 GI burden was significantly higher in both C3^−/−^ and C4^−/−^ mice at 48 h, and by 96 h was >100-fold increased over WT mice (Fig. S4A). As shown in Fig. S4B, at 24 h post-challenge, MKP103 also translocates from the GI tract to extraintestinal sites, predominantly to the liver in WT and C3^−/−^ mice, and additionally to the spleen in C3^−/−^ mice. Despite this dissemination, all mice survived (Fig. S4C), indicating that MKP103 was efficiently cleared in the periphery, consistent with reports of its limited virulence in mice (30, 44–46). Thus, in contrast to its limited role in controlling *Kpn* colonization and extraintestinal dissemination following natural acquisition, in the context of antibiotic-induced *Kpn* supercolonization, C4 is required along with C3 to limit bacterial burden in the GI tract for both hypervirulent and classical strains.

### *Kpn* GI colonization level correlates with *Kpn* dissemination burden in the liver in WT and C4^−/−^ mice

**Fig 8:**
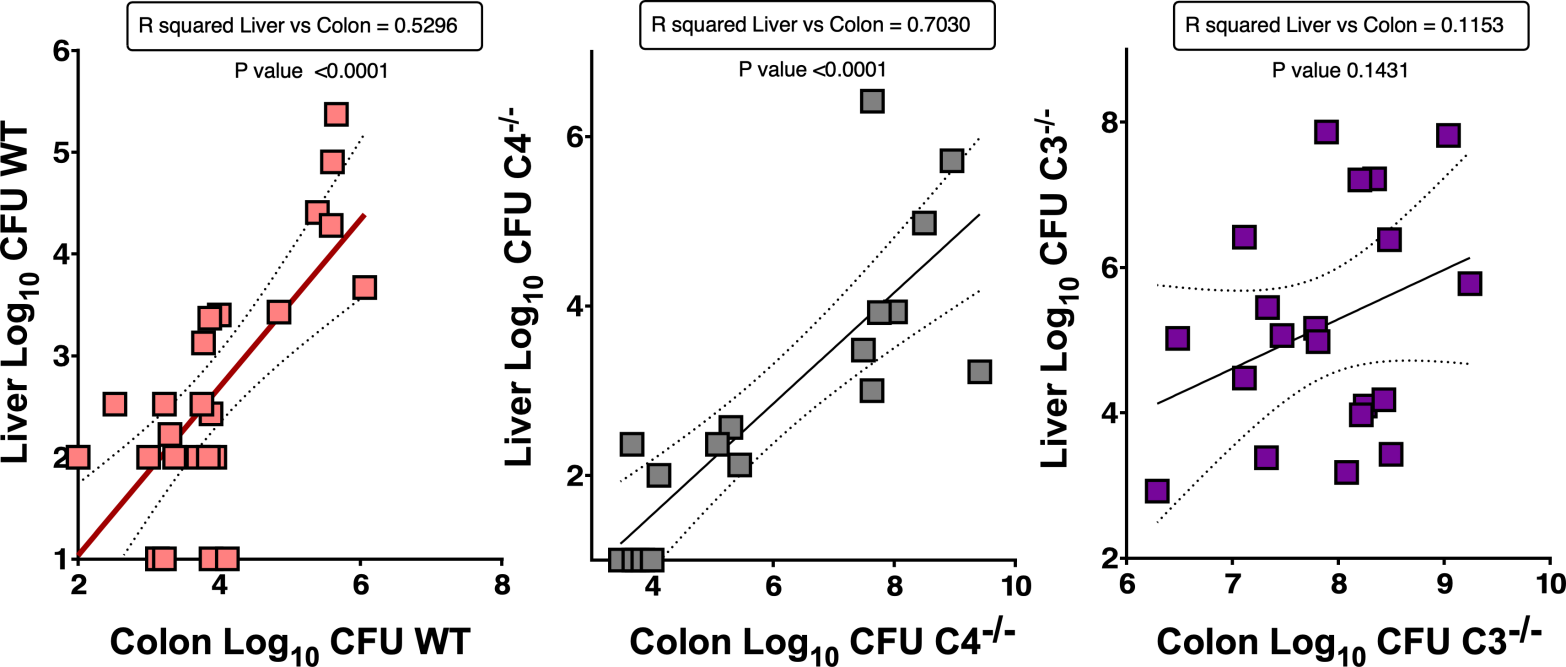
*Kpn* burden in the GI tract correlates with the extent of bacterial dissemination to the liver. Correlation analyses of KPPR1S burden in the GI tract (colon) and liver at 24 h post-infection in WT, C4^−/−^, and C3^−/−^ mice, displayed as a simple linear regression curve. *R*^2^ for the linear regression is listed, with data points representing individual mice (*n* ≥ 17/group). Pooled results from ≥4 independent experiments.

To assess whether the level of GI colonization was associated with the degree of systemic dissemination, we examined the relationship between *Kpn* burden in the GI tract and liver via correlation analysis (Fig. 8). In KPPR1-infected WT mice, there was a significant correlation between colon and liver burdens (*R*² = 0.53, *P* < 0.0001). This association was even stronger in C4^−/−^ mice (*R*² = 0.71, *P* < 0.0001). The correlation in C3^−/−^ mice was near-significant but weak (*R*² = 0.10, *P* = 0.1), possibly due to the excessive GI burden combined with the general lack of containment upon dissemination to the liver. Thus, under both a WT setting as well as in C4 deficiency, there is a clear association between *Kpn* burden in the GI tract and the extent of dissemination to the liver.

## DISCUSSION

The complement system, an evolutionarily ancient component of the innate immune response, plays a fundamental role in protecting the host against microbes that breach barriers and enter the bloodstream (47, 48). However, emerging evidence points to a role for complement production at these barrier surfaces, including the GI and respiratory tracts (17, 49–55). The data presented herein reveal several insightful findings regarding the role of complement in *Kpn* pathogenesis. First, we reveal an unexpected role for locally produced complement proteins C3 and C4 in their ability to limit *Kpn* colonization and expansion within the GI tract. Second, we demonstrate that despite its ability to prevent lysis, the K2 capsule produced under GI-mimetic conditions allows for C3 deposition to occur via the alternative pathway, and although C4b deposition is present along with C3b deposition on *Kpn* in the gut, only C3 is required to control GI burden following natural colonization. Third, besides providing protection against systemic disease, C3 is critical for controlling GI burden, likely through its role in promoting phagocyte recruitment in conjunction with its capacity for opsonization. Finally, we find C4—central to the classical and lectin pathways—is dispensable for controlling GI burden and dissemination at low colonizing burdens encountered in the absence of selective antibiotics; however, under conditions of antibiotic-induced *Kpn* bloom in the gut, C4 becomes critical for controlling GI burden. As *Kpn* GI burden correlates with the degree of translocation and dissemination (Fig. 8), complement-mediated control of GI burden, whether it be C3-dependent/C4-independent in the context of natural colonization or C3- and C4-dependent in the context of antibiotic treatment, is a critical first line of defense against *Kpn* systemic disease.

The finding that C3 and C4 proteins were present in fecal samples in naïve mice is consistent with the findings reported by Wu et al., which revealed the presence of transcripts for C3 and C4 in colonic leukocytes and stromal cells under homeostatic conditions (21). The increase in C3 and C4 levels in the lumen is also consistent with the mild inflammatory response induced by *Kpn* colonization, as evidenced by upregulation of lipocalin-2 levels (Fig. S1C). These proteins, along with C1q, C2, and factors D and P, were shown to be transcriptionally upregulated in response to *C. rodentium*, supporting the potential for both classical- and alternative pathway-mediated opsonophagocytosis to occur in the GI tract. Whether oral exposure and/or oropharyngeal colonization impacts the *Kpn* gut response, as has been observed in some disease states (56), requires further investigation. Additional investigation is required to identify the sources of complement proteins in the context of *Kpn* colonization. Indeed, myeloid cell recruitment may be a key feature of the coordinated upregulation of complement proteins required for both classical and alternative pathways in response to GI bacteria.

Our finding that C3 restricts *Kpn* gut burden aligns with results obtained for *Citrobacter rodentium* infection (21). However, while the Wu et al. study suggested infection-induced neutrophil recruitment induces mucosal C3 production and is associated with alternative pathway-mediated phagocytosis, our data directly demonstrate C3 as being critical for initial recruitment of phagocytic cells to the infection site and thereby suggest a broader role for C3 in defense of the GI tract in the context of *Kpn* infection. While we did not identify the source of mucosal C3, the initial low levels, likely produced by colonic stromal cells in naïve mice (21), may be critical for myeloid cell recruitment, as is evidenced by the reduced number of Gr1^+^ cells isolated from colons of non-infected C3^−/−^ mice. The mechanisms by which C3 promotes the recruitment of myeloid cells in the gut are not yet clear. While C3a can function as a mild anaphylatoxin, it also has the potential to stimulate local cells, which may affect recruitment through other signals. The extent to which local C3a-responsive cells, such as mast cells and/or epithelial cells, as well as *Kpn* LPS-induced upregulation of C3aR on these and other cell types (57), are involved in eliciting signals that may be required for myeloid cell recruitment requires further study. We only identified weak shifts in green fluorescent signals in support of phagocytosis of GFP^+^ *Kpn* by myeloid cells isolated from the colons of infected mice, perhaps due to the loss of GFP signal within acidic phagolysosomes (Fig. S1H); nonetheless, we detected C3 and C4 deposition on *Kpn* within fecal samples, indicating opsonization does occur within the gut lumen. While this supports the potential for opsonophagocytosis to occur, this remains to be formally demonstrated.

The requirement for C4 in controlling GI colonization in the context of high bacterial burden associated with antibiotic use represents a novel and significant finding. The increase in C4 in fecal samples, along with detection of C4 deposition on GI-derived *Kpn,* supports the possibility that C4 functions to promote opsonization either directly (CR1 is expressed by murine M1-polarized macrophages and supports C4b-mediated opsonophagocytosis in some cases [58]) or through promoting C3 convertase formation and hence C3b deposition in synergy with the alternative pathway C3 convertase. While C4 does not appear to be critical for myeloid cell recruitment, its importance during antibiotic-induced high colonizing burden suggests a need for additional pathways of C3b deposition to effectively control elevated bacterial loads. It is unclear how C4b deposition occurs *in vivo*, although C1q has been reported to bind to *Kpn* porins independently of antibody binding (59), supporting the possibility of an antibody-independent, C4-dependent pathway of activation. This represents an important area of investigation for the future.

The critical role for C3 relative to C4 in regulating dissemination may stem partly from its role in controlling GI burden. C3b deposition by the alternative pathway is likely critical both in the GI tract and in the circulation, as supported by our CVF depletion results, and the increased bacterial burden and susceptibility of C3^−/−^ mice to systemic challenge relative to C4^−/−^ mice. Importantly, our CVF depletion results suggest a critical role for circulating C3 in limiting systemic *Kpn* dissemination, distinct from its role in controlling GI burden. Expression of C3b receptors (CRIg, CD11b/CD18) by Kupffer (KC) and other phagocytic cells provides a likely path toward *Kpn* clearance (60, 61). CRIg is the major C3 receptor on KCs responsible for clearance, although unknown scavenger receptor(s) appear to also be involved. This may explain the lack of morbidity in C3^−/−^ mice found with the classical MKP103 strain in our study. Indeed, there are differences in *Kpn* strains that associate with capsule type that may lead to C3- and C4-independent clearance by the liver. This was elegantly shown by Huang et al. (27) whereby a K2 capsule switch to K23 resulted in a CRIg-dependent but C3-independent mechanism of liver clearance. Although circulating *Kpn*-reactive natural antibodies may facilitate clearance, the limited role observed for the C4-dependent classical pathway under natural conditions in this study suggests this defense mechanism may be secondary to the alternative pathway, although adaptively acquired antibodies likely invoke this pathway along with complement-independent Fc receptor-mediated clearance mechanisms. Importantly, the induction of the latter pathway could explain discrepancies in the literature regarding the role C3 plays in defense against *Kpn* (KPPR1) dissemination.

Our findings reveal hierarchical and context-dependent roles of the immune system in managing GI burden, where the need to preserve a diverse commensal microbiota must be balanced with effective barrier surveillance and pathogen clearance. While C3 remains a central effector across conditions, following antibiotic-induced dysbiosis and the emergence of a supershedder phenotype with high bacterial burden, C4 becomes essential. Given that *Kpn* is a leading source of hospital-acquired infections (62), work of this nature is pivotal for devising strategies to limit colonization and dissemination events within susceptible patients and to mitigate transmission and disease outbreaks. These insights highlight the dynamic nature of host defense during *Kpn* infection and may inform therapeutic strategies aimed at modulating complement activity to strengthen mucosal immunity.

## MATERIALS AND METHODS

### Mice

WT C57BL/6, C3^−/−^ (B6.129S4-C3tm1Crr/J), and C4^−/−^ (B6.129S4-C4btm1Crr/J) mice were obtained from Jackson Laboratory. C4^−/−^ mice were further backcrossed four generations onto the C57BL/6 background.All mice were sex and age (8–10 weeks) matched, housed under sentineled SPF conditions, andacclimated to BSL2 housing 24 hours prior to infections.

### Infections

Oral infections were performed for sex- and age-matched cagemates of each genotype (*n* = 4–5/group) at the same time to limit confounding factors, as described by Young et al. (30). No randomization or blinding was applied. Mice were fasted for ~4 h, then given ~10⁶ CFU of streptomycin-resistant *Kpn* (KPPR1S [63, 64]) in 2% sucrose-PBS, via two 50 µL doses 1 h apart (65). For antibiotic-induced supercolonization, antibiotics were provided in drinking water three days prior to inoculation—streptomycin sulfate (500 mg/L) for KPPR1S infections and ampicillin (250 mg/L) for MKP103 (43). Antibiotics were provided between days −3 and 7 of infection, unless otherwise noted. After 4 h fasting, mice received 10⁷ CFU of KPPR1S or MKP103 in 100 µL 2% sucrose-PBS via 20-gauge gavage. Feces and tissues were homogenized in PBS, centrifuged, and supernatants were serially diluted and plated on selective agar to assess bacterial burden (65). In some experiments, mice were orally inoculated with KPPR1S and subsequently provided antibiotic-containing drinking water to promote the development of a supershedder phenotype.

### CVF treatment of WT mice

A dose of 12.5 µg CVF per 25 grams of body weight was given to WT mice via intraperitoneal injection (21) 1 day before infection and on days 1, 3, 5, and 7 after infection. Control mice received PBS injections.

### Microbiota normalization via co-housing of WT and C3^−/−^ weanlings prior to KPPR1S infection

To promote microbiome homogenization, WT and C3^−/−^ weanling mice were co-housed beginning at weaning (postnatal day 21). Mice were mixed in the same cages in equal numbers per genotype and maintained together for 3 weeks prior to infection experiments.

### ELISAs

Fecal supernatants (100 mg/mL in PBS) were centrifuged at 14,000 × *g* for 10 min at 4°C and stored at –20°C. C3, C4, and lipocalin-2 levels were measured using ELISA kits (Abcam ab157711; Hycult HK217; Mouse Lipocalin-2/NGAL DuoSet ELISA).

### *In vitro* complement killing and deposition assay

To assess complement-mediated killing, *K. pneumoniae* strains (KPPR1S and ΔwcaJ), grown in LB or cecal filtrate (66, 67), were pelleted and resuspended in PBS to a final concentration of 4 × 10⁹ CFU/mL. Bacteria (~2 × 10⁷ CFU in 5 µL), DMEM (45 µL), and 5 µL of complement source were combined. Complement sources included rabbit complement (Cedarlane #CL3111), fresh WT mouse serum, and human serum depleted of antibodies (Pel-Freez Biological #34010). In parallel, heat-inactivated (56°C for 30 min) and EGTA-treated (10 mM EGTA + 5 mM MgCl₂) versions of each complement source were used to control for complement-independent effects and to selectively inhibit classical and lectin pathways. After 30 min of incubation at room temperature, bacterial survival was quantified by serial dilution and plating on LB agar. To quantitate C3b deposition, KPPR1S was labeled with 1 µM CellTracker Violet (30 min), washed in PBS + 1% BSA, and incubated with 10% rabbit complement, heat-inactivated complement, or PBS in RPMI at 37°C for 1 h. After washing, bacteria were stained with goat anti-C3/C3b F(ab′)_2_-FITC (25 min), washed, and fixed in 1% buffered formalin. Gating was based on size (Apogee beads) and CTV^+^ signal. Background staining was determined using goat IgG F(ab′)_2_-FITC (Fig. S2C).

### Generation of and detection of GFP-expressing *K. pneumoniae* strain KPPR1S

To facilitate identification of *Kpn* during flow cytometric analysis of complement activation *in vivo*, a GFP-expressing KPPR1S strain was transformed via electroporation with the plasmid pJH026, which encodes GFP under the control of the constitutive Pem7 promoter in the pPROBE vector (68). Transformants were selected on LB agar containing kanamycin (50 µg/mL). Kanamycin was maintained in culture media to ensure plasmid retention during *in vitro* growth prior to infection. This strain was used in oral infection experiments to assess complement deposition on KPPR1S in the GI tract. To promote supercolonization and facilitate bacterial identification, streptomycin sulfate (500 mg/L) was added to the drinking water 72 h post-inoculation. Fecal samples were harvested at 96 h following oral inoculation, weighed, and homogenized in sterile PBS. Bacteria were isolated by filtration and centrifugation and washed prior to staining. Recovered bacteria were incubated with biotinylated rat anti-mouse C3b (11H9) and rat anti-mouse C4b (16D2). A biotinylated rat IgG was included as a negative control to account for non-specific binding. Samples were washed and incubated with streptavidin-conjugated fluorophores and analyzed by flow cytometry.

### Phagocyte recruitment

Phagocyte recruitment was assessed 48 h post-infection. After perfusion with 30 mL PBS, colons were excised, flushed, and digested in collagenase IV (1  mg/mL) and DNase I (5 U/mL) in PBS containing 2% newborn calf serum (NCBS) at 37 °C for 20–30 min with agitation, then mechanically disrupted and filtered through 70 μm mesh. Cells were pelleted (400 × *g*, 10 min), resuspended in PBS-2% NCBS containing Countbright beads, CD11b-BV650, CD45-AF700, GR1-AF647, and Live/Dead Aqua (25 min). After washing and fixation (1.5% formalin), samples were acquired on a Fortessa X20 and analyzed using FlowJo software.

### Gut microbiome analysis

Gut microbiome profiles were assessed as per our previously described methods (30, 67, 69, 70). Briefly, the fecal samples were preserved at −80°C until microbial DNA extraction. Genomic DNA was extracted from a 200 mg fecal specimen using the QIAamp PowerFecal Pro DNA Kit (Qiagen Inc.) following the manufacturer’s instructions. The hypervariable V4 region of the bacterial 16S rRNA gene was PCR-amplified with the universal primers 515F/806R, as per the Earth Microbiome Project benchmark protocol (71). The resulting amplicons were purified utilizing AMPure magnetic purification beads (Agencourt) and quantified using a Qubit 4 fluorimeter (Invitrogen). Libraries with equal molar concentrations were combined into a single pool, forming the final amplicon library, which was subjected to paired-end (2 × 300  bp) sequencing via an Illumina MiSeq sequencer (utilizing MiSeq reagent kit version 3; Illumina). The resultant raw sequencing data underwent processing using QIIME2-DADA2 (version 2-2023.5) bioinformatics pipeline (72). Initial QC steps involved demultiplexing and quality-filtering of raw sequences by employing the q2-demux plugin, followed by trimming and denoising steps through the DADA2 workflow. Subsequently, all identified amplicon sequence variants were aligned via MAFFT and were subjected to taxonomy assignment using the sklearn classifier along with the pretrained naïve Bayes taxonomy classifier, aligned against the 99% SILVA 138 database. Alpha-diversity analysis utilized the Shannon index (for richness and evenness). For beta-diversity analysis, the Bray-Curtis dissimilarity index was applied, with the results visualized through principal coordinate analysis. Statistical analyses, including nonparametric Kruskal-Wallis tests and PERMANOVA with 999 random permutations, were applied to detect significant differences in microbial diversity and structure. Differential abundance of bacterial taxa was identified using the LEfSe biomarkers discovery algorithm that applies a nonparametric Kruskal-Wallis test to detect significant differences among groups, followed by a pairwise Wilcoxon rank-sum test to evaluate pairwise significance, and ultimately performs a LDA to estimate the effect size and rank the microbial features (73). The visualization of results was carried out using “R” and “Python” packages. The raw microbiome sequencing data that support the findings of this study are openly available in National Center for Biotechnology Information-Sequence Read Archive at https://www.ncbi.nlm.nih.gov/sra, under reference number PRJNA1328802.

### Statistical analysis

Statistical analyses were performed using GraphPad Prism v.10.2.3, with graphing of individual data to assess assumptions where parametric tests were used. Two-group comparisons used the Mann-Whitney *U*-test. Multiple groups were analyzed by Kruskal-Wallis with Dunn’s test, one-way ANOVA with Bonferroni correction, or two-way ANOVA with appropriate *post hoc* tests. Survival was analyzed using the log-rank (Mantel-Cox) test.

## Data Availability

All data generated and analyzed are included in this published article and its supplemental information. Raw data generated for the current study are available at NCBI-SRA under PRJNA1328802.

## SUPPLEMENTAL MATERIAL

Supplemental material mbio.03416-25-s0001.pdf Figures S1-S4 and Table S1.

ASM does not own the copyrights to Supplemental Material that may be linked to, or accessed through, an article. The authors have granted ASM a non-exclusive, world-wide license to publish the Supplemental Material files. Please contact the corresponding author directly for reuse.
