# Supplementary material for: Divergent roles for complement components C3 and C4 in controlling Klebsiella pneumoniae gut colonization and systemic dissemination
Source: mBio. 2026 Feb 11;17(3):e03416-25. doi: 10.1128/mbio.03416-25 (PMC12977614; doi:10.1128/mbio.03416-25)
Supplement: Supplemental material — Figures S1-S4 and Table S1. [file mbio.03416-25-s0001.pdf]

Supplemental Figures and Legends

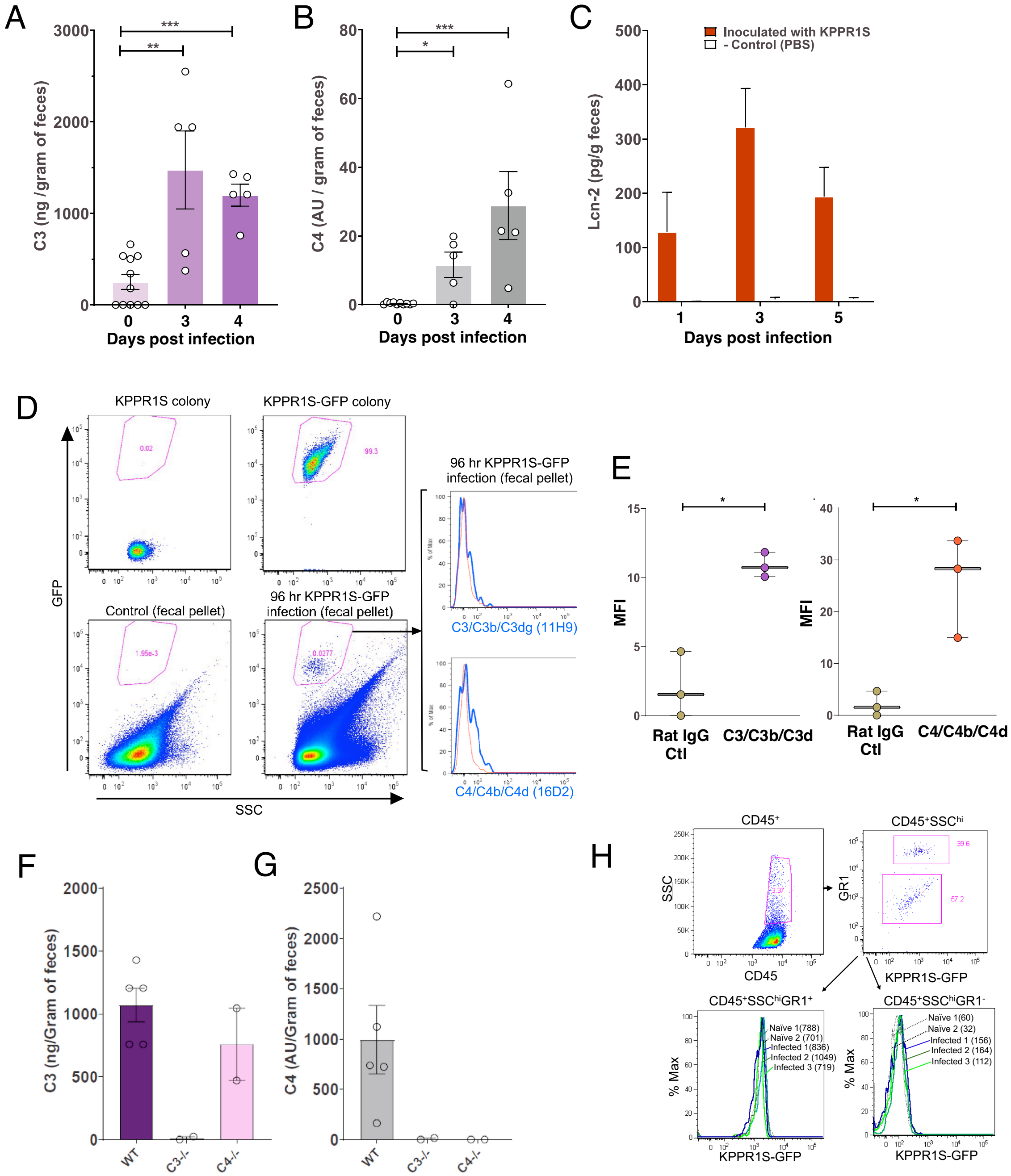

Figure S1. Oral *Kpn* inoculation elevates fecal complement C3, C4, and lipocalin-2 levels, and C3/C4 deposition is present on GFP-tagged *Kpn* isolated from the gut. A–B) C3 (A) and C4 (B) levels were measured by ELISA in fecal samples from C57BL/6J WT mice following oral inoculation with *K. pneumoniae* strain KPPR1S (~10^6 CFU). Each symbol represents an individual mouse (n ≥ 5 per group). Statistical significance was assessed by one-way ANOVA with Bonferroni post hoc test. *p < 0.05, **p < 0.01, ***p < 0.001, ****p< 0.0001. **C)** Fecal lipocalin-2 (Lcn-2) levels measured by ELISA in WT mice following oral inoculation with KPPR1S (~10^6 CFU; n ≥ 3 per group) or PBS. **D-E)** GFP-expressing KPPR1S was generated by introducing plasmid pJH026 (Pem7-GFP in pPROBE) to assess C3 and C4 deposition levels in vivo. In D, detection of GFP-expressing KPPR1S by flow cytometry is shown for colony-grown and KPPR1S isolated from fecal pellets, along with C3/C3b/C3d and C4/C4b/C4d deposition as detected by 11H9 and 16D2 mAb detection, respectively, relative to rat IgG isotype staining, with MFI quantitation shown in panel E. Points represent individual mice (*p < 0.05, Mann Whitney U test). **F-G)** Detection of C3 (F) and C4 (G) levels in fecal supernatants from WT, $C3^{-/-}$, and $C4^{-/-}$ mice 96 hours post oral KPPR1S inoculation (~10^6 CFU, n ≥ 2 per group) by ELISA. **H)** GFP signal in colonic $CD45^+SSC^{hi}$ $GR1^+$ and $GR1^-$ granulocytes, with MFIs indicated for individual mice. In A-C, F, and G, mice were orally infected with KPPR1S without antibiotics. In D-E and H, mice were orally infected with GFP^+ KPPR1S without antibiotics and at 72 hours, streptomycin was added to drinking water for 24 hours to enable detection of higher numbers of GFP^+ *Kpn* at 96 hours.

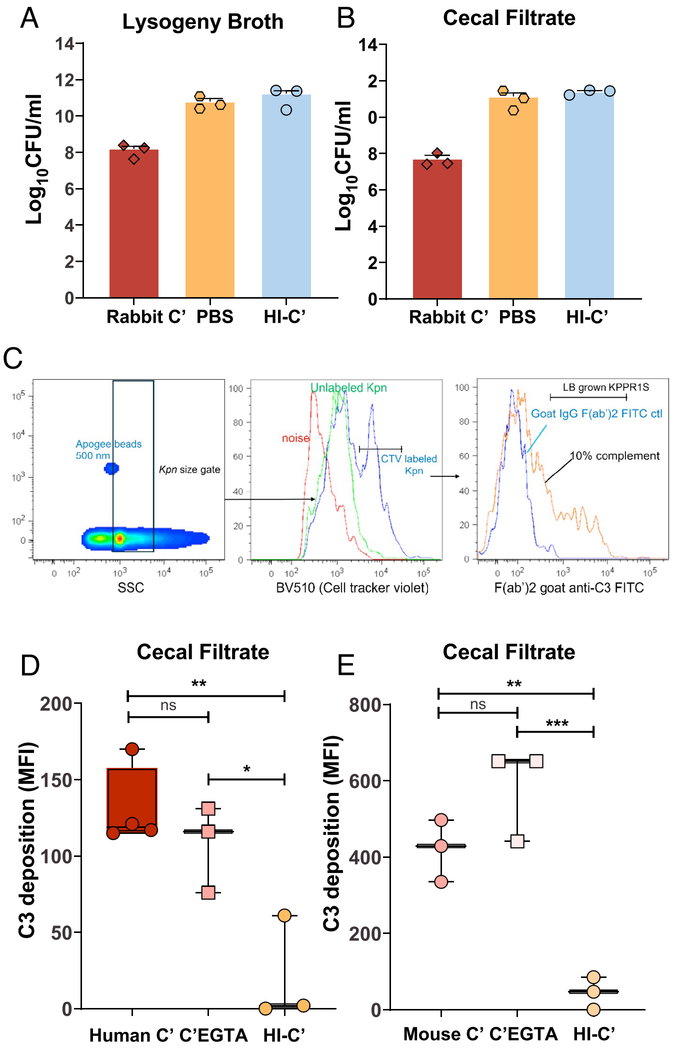

Figure S2. Effects of complement pathways on *Kpn* in vitro.

A–B) An isogenic capsule-deficient KPPR1S strain (ΔwcaJ) grown in LB or cecal filtrate was incubated for 30 minutes at 37 °C in with 10% rabbit complement (C'), heat-inactivated complement (HI-C'), or PBS. **C-E)** Detection of C3b deposition on *in vitro*-grown KPPR1S. *Kpn* was labeled with 1 uM Cell Tracker Violet for 30 minutes, washed with PBS containing 1% BSA, and incubated with C' in RPMI at 37°C for 1 hour. Bacteria were washed in PBS containing 1% BSA followed by staining with goat anti-C3/C3b $F(ab')_2$-FITC for 25 minutes. Bacteria were identified based on size using selective gating with Apogee beads followed by gating Cell tracker violet (CTV) positive bacteria. Nonspecific goat IgG $F(ab')_2$-FITC was used to determine background staining. Mean fluorescent intensities (MFI) of C3b (FITC) signals were determined as a measure of C3b deposition level. C) Flow cytometry gating strategy to identify CTV-labeled KPPR1S based on size, CTV positivity, and negative control (goat IgG $F(ab')_2$ FITC) staining relative to $F(ab')_2$ goat anti-mouse C3 FITC staining. D) C3b deposition on KPPR1S cultured in cecal filtrate was quantified by flow cytometry after 1-hour incubation at 37 °C using either human (D) or mouse (E) C', EGTA-treated C', and HI-C' controls. Statistical analysis: one-way ANOVA with Bonferroni post hoc test ($n \geq 3$ per group). $*P < 0.05$, $**P < 0.01$, $***P < 0.001$, $****P < 0.0001$.

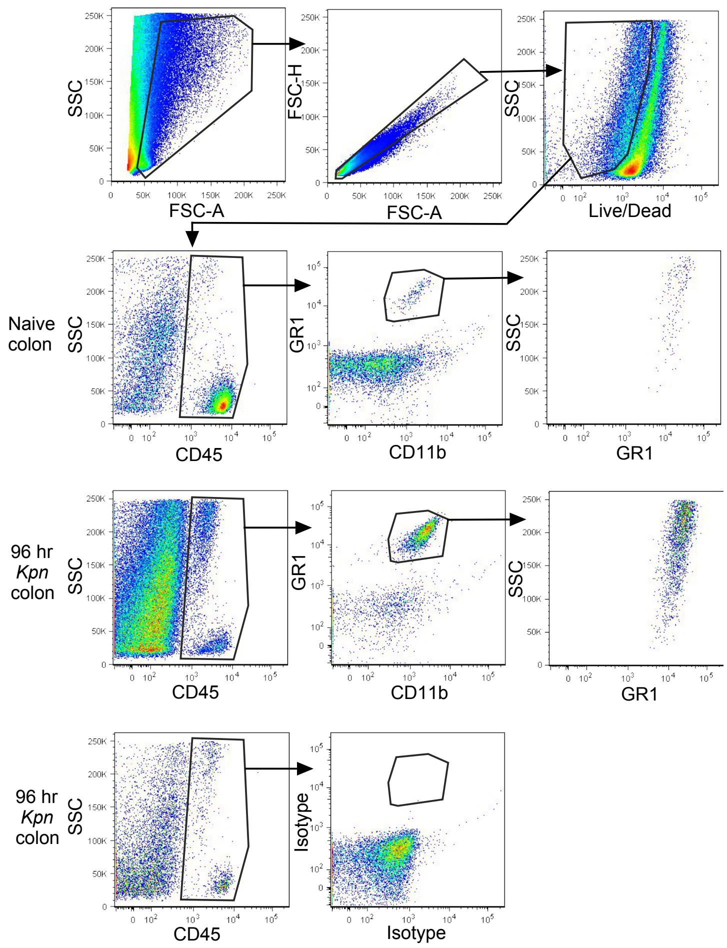

Figure S3. Flow cytometric gating strategy to identify colonic leukocytes.
Processed colons were stained for CD45, CD11b, GR1, and live/dead exclusion. Cells were gated as indicated in the upper panel, excluding debris and dead cells. Live cells were gated for CD45 expression, followed by $GR1^{+}CD11b^{+}$ staining. Representative staining for colons from naïve and infected mice (96 hours post oral infection) is shown, along with isotype control staining for $CD45^{+}$ cells gating in infected colon cell suspensions.

Supplemental Figures and Legends

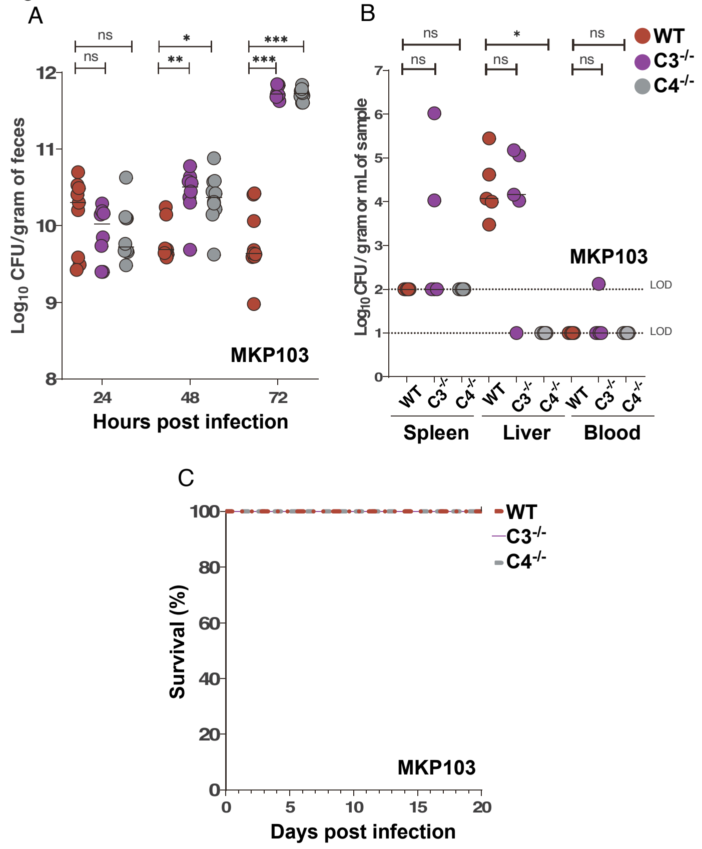

Figure S4. Bacterial burden and survival of *Kpn* classical strain MKP103 in WT, C3$^{-/-}$, and C4$^{-/-}$ mice following antibiotic pretreatment.

A) Fecal shedding at 24, 48, and 72 hours post-intragastic gavage with MKP103 in mice pretreated with ampicillin (250 µg/mL in drinking water from d-3 to 7 post infection). **B)** Bacterial burden in spleen, liver, and blood at 24 hours after intragastric challenge. Panel A and B, each point represents an individual mouse; dotted line indicates the limit of detection (L.O.D.). **C)** Kaplan–Meier survival curves of WT (red), C3$^{-/-}$ (purple), and C4$^{-/-}$ (gray) mice following oral gavage. All strains were grown overnight in LB; inoculum ~10^7 CFU. Statistical tests: Kruskal–Wallis with Dunn's post hoc (A–B) and Log-rank (Mantel–Cox) (C). n ≥ 5 per group. *$P < 0.05$, **$P < 0.01$, ***$P < 0.001$, ****$P < 0.0001$.

Supplemental Figures and Legends

Table I. Survival Analysis

**Genotype**	**Mice (live/total)**	**Survival (%)**	**Fisher's Exact Test *p value***
WT	10/10	100	<0.0001
$C3^{-/-}$	0/10	0	
WT	10/10	100	0.0054
$C4^{-/-}$	4/10	40	
$C3^{-/-}$	0/10	0	0.0433
$C4^{-/-}$	4/10	40	

Supplemental Table I. Survival of WT, $C4^{-/-}$ and $C3^{-/-}$ mice following systemic challenge with *Kpn*

WT, $C3^{-/-}$, and $C4^{-/-}$ mice were infected intraperitoneally with ~100 CFU of KPPR1S strain to assess susceptibility to systemic infection. Survival outcomes mirrored those observed during gastrointestinal infection: WT mice showed the highest survival, $C4^{-/-}$ mice had intermediate survival, and $C3^{-/-}$mice were the most susceptible. Statistical significance was determined using one-tailed Fisher's exact test (10 mice/group).
